# Bridging the gaps in alzheimer’s disease: a comprehensive review of current and emerging therapies

**DOI:** 10.1007/s10787-026-02151-3

**Published:** 2026-03-23

**Authors:** Rasha E. Mostafa, Gihan F. Asaad

**Affiliations:** Pharmacology Department, Medical Research and Clinical Studies Institute, National Research Centre, 33 ELBohouth St. (former EL Tahrir St.), P.O. 12622, Dokki, Cairo, 60014618 Egypt

**Keywords:** Alzheimer’s disease, Neurodegeneration, Amyloid-beta, Tau protein, Neuroinflammation, Therapeutic strategies, Disease-modifying treatments

## Abstract

Alzheimer’s disease (AD) is a progressive neurodegenerative disorder and the leading cause of dementia worldwide, accounting for 60–80% of dementia cases. It is characterized by gradual cognitive decline, neuronal loss, and pathological hallmarks, including amyloid-β (Aβ) plaques and neurofibrillary tangles of hyperphosphorylated tau protein. This review provides a comprehensive overview of AD, emphasizing its etiology, molecular mechanisms, risk factors, and current therapeutic strategies. Multiple hypotheses, including the amyloid cascade, tau, cholinergic, vascular, and neuroinflammatory theories, are discussed to elucidate disease pathogenesis. Genetic mutations in the APP, PSEN1, and PSEN2 genes, along with environmental and lifestyle factors, are shown to influence disease onset and progression. The treatment landscape is rapidly evolving from traditional symptomatic therapies, such as cholinesterase inhibitors and NMDA receptor antagonists, to emerging disease-modifying agents targeting amyloid, tau, and neuroinflammation. Novel approaches, including glutaminyl cyclase inhibitors, PDE inhibitors, serotonin receptor modulators, and metabolic therapies, offer new hope for altering disease progression. Non-pharmacological interventions, such as diet, exercise, and lifestyle modifications, also play a key preventive role. Despite ongoing challenges, advancements in biomarker research, neuroimaging, and precision medicine are improving early detection and individualized treatment strategies. Continuous innovation in pharmacotherapy and diagnostics promises to reshape the future of AD management and enhance patients’ quality of life. The current review focuses on bridging the gaps in AD’s current and emerging Therapies.

## Introduction

Alzheimer’s disease (AD) is a progressive neurodegenerative disorder that represents the most common cause of dementia in older adults, accounting for an estimated 60–80% of cases worldwide. First described by Dr. Alois Alzheimer in 1906, the disease is clinically characterized by a gradual decline in cognitive functions, including memory, reasoning, language, and the ability to perform even basic daily tasks. With the rapid aging of populations globally, Alzheimer’s disease has become a major public health concern, affecting over 55 million people and placing a substantial emotional and economic burden on families, caregivers, and healthcare systems (Kepp et al. 2023).

Pathologically, AD is characterized by the accumulation of two main proteins in the brain: amyloid-beta (Aβ) plaques and neurofibrillary tangles made of hyperphosphorylated tau protein. These hallmark features are linked to widespread neuronal loss, synaptic dysfunction, and inflammation, which contribute to the progressive cognitive decline observed in affected individuals. Although the exact mechanisms behind the disease are not fully understood, increasing research indicates that a complex interplay between genetic, environmental, and lifestyle factors plays a role in its onset and progression (O’Brien and Wong 2011a, b).

Over the past several decades, significant advances have been made in understanding the molecular and cellular basis of AD. Genetic studies have identified both rare, early-onset familial forms of the disease linked to mutations in certain genes, such as APP, PSEN1, and PSEN2, as well as common risk alleles like APOE ε4 associated with late-onset AD (Granzotto and Sensi 2024).

Advances in neuroimaging, biomarker discovery, and systems biology have enhanced the ability to detect and monitor AD in its earliest stages, often before the appearance of clinical symptoms (Kurkinen et al. 2023).

Despite these developments, effective treatment options remain limited. Current therapies, such as cholinesterase inhibitors and NMDA receptor antagonists, offer only modest symptomatic relief without altering disease progression. However, recent breakthroughs in disease-modifying therapies targeting amyloid pathology, such as monoclonal antibodies, have renewed hope for slowing the disease course. These advancements highlight the urgency of continued research and innovation in diagnostics, therapeutics, and care strategies (Selkoe and Hardy 2016a, b).

This review article aims to provide a comprehensive overview of AD, including its etiology, epidemiology, clinical presentation, pathophysiology, genetic underpinnings, current treatment landscape, gaps, and future directions in research.

## Etiology and molecular underlying mechanisms

Alzheimer’s disease (AD) is characterized by progressive cognitive deterioration and memory impairment. The etiology involves a complex interaction of genetic, physiological, environmental, and behavioural factors that alter normal brain function. Molecular diseases such as amyloid-β plaques and neurofibrillary tangles of hyperphosphorylated tau result in synaptic loss, inflammation, and neuronal dysfunction. Formulating successful treatment options and tracing the origin of the disease rely on an understanding of these fundamental mechanisms. Rather than being a methodically applied categorization system, the AT(N) framework is provided in this review as a conceptual biological model to contextualize the three main pathological aspects of Alzheimer’s disease: amyloid pathology, tau pathology, and neurodegeneration. Studies are examined based on their major pathogenic focus without formal segmentation into AT(N) categories due to the narrative breadth of this study and the heterogeneity of the evaluated literature.

### Amyloid cascade hypothesis

The early 1990s idea argues that amyloid-β (Aβ) peptide buildup, particularly the 42-amino acid variant Aβ₄₂, produces a neurological cascade that leads to Alzheimer’s symptoms. Over the past 30 years, this paradigm has transformed our understanding of Alzheimer’s disease etiology and led to new treatments Transmembrane proteins in different organs, including the brain, degrade amyloid precursor protein (APP) and produce amyloid-β peptides. Two pathways can process APP in normal physiological conditions: the amyloidogenic pathway, which produces Aβ peptides by cleaving β-secretase (BACE1) and γ-secretase, and the non-amyloidogenic pathway, which inhibits Aβ production by cleaving α-secretase within the Aβ domain. Due to its tendency to misfold and collect, Aβ₄₂ is more damaging than the shorter isoform (O’Brien and Wong 2011a, b). The excessive or inadequate clearance of Aβ₄₂ causes its accumulation in the extracellular space, forming soluble oligomers, protofibrils, and insoluble plaques, a hallmark of Alzheimer’s disease. Oligomers of Aβ are more dangerous than plaques, as they affect synaptic function, change calcium homeostasis, cause oxidative stress, and activate microglia and astrocytes (Selkoe and Hardy 2016a, b). Mutations in the APP or PSEN1 and 2 genes, which encode γ-secretase complex components, are linked to early-onset AD and increase Aβ₄₂ production. Down syndrome patients, who have an extra copy of chromosome 21 carrying the APP gene, are more likely to acquire amyloid pathology and dementia in middle age (Weggen and Beher 2012; Castellani et al. 2024). There is now a consensus that the amyloid hypothesis can interact with a complex network of pathologies, including tau hyperphosphorylation, neuroinflammation, mitochondrial dysfunction, and vascular impairment (Gericke et al. 2023; Behl 2024).

The amyloid cascade is not a strictly linear process; rather, there is a dynamic interplay between Aβ accumulation and tau pathology, neuroinflammation, synaptic dysfunction, vascular changes & mitochondrial impairment. These mechanisms create linked positive feedback loops that can accelerate the disease course. Rather than being the only upstream driver, Aβ may be an initiating or accelerating factor. Moreover, the Aβ production and clearance can be affected by downstream pathologies. This multifactorial approach more accurately reflects the current understanding of AD pathophysiology (Mekala and Qiu 2025).

### Tau hypothesis

Neurofibrillary tangles (NFTs) of abnormally phosphorylated tau protein indicate neuronal dysfunction and cognitive decline in Alzheimer’s disease. Neurons’ tau protein stabilizes microtubules, which are essential for structural integrity and intracellular trafficking. Healthy brain phosphorylation affects Tau’s microtubule affinity. Tau hyperphosphorylates, detaches from microtubules, and aggregates into insoluble paired helical filaments (PHFs) in Alzheimer’s disease, forming neurofibrillary tangles. These tangles inhibit microtubule function, causing axonal transport, synaptic dysfunction, and neuronal death (Guo et al. 2017, 2022; Arnsten et al. 2021). The degree of clinical symptoms in Alzheimer’s disease is correlated with tau pathology, while amyloid-β pathology shows early and diffusely in asymptomatic individuals. Tau disease follows a methodical progression from the entorhinal cortex and hippocampus to the neocortex, according to research. Mutations in the MAPT gene, which encodes tau protein, cause frontotemporal dementia (FTD), a neurological illness similar to Alzheimer’s. Tauopathies such as corticobasal degeneration and progressive supranuclear palsy show tau pathology beyond Alzheimer’s disease, highlighting its importance in neurodegeneration (Ye et al. 2023a, b; Nasb et al. 2024). Hyperphosphorylated tau causes mitochondrial malfunction, oxidative stress, synaptic loss, glial cell activation, and microtubule dynamics disorder, causing cerebral damage and inflammation. The pathology of tau may interact with other components of Alzheimer’s disease, such as amyloid-β. Aβ accumulation can activate GSK-3β, leading to disease progression and tau hyperphosphorylation (Dominguez-Gortaire et al. 2025; Valiukas et al. 2025).

Tau phosphorylation can distinguish between early and late-stage AD as tau experiences site-specific, mild phosphorylation in the early stages of AD, which interferes with synaptic signalling and neuronal plasticity without causing significant aggregation. These early alterations are linked to mild cognitive impairment and are frequently reversible. Microtubule binding is lost as the disease worsens due to extensive hyperphosphorylation of tau at several epitopes. Hyperphosphorylated tau aggregates into mature neurofibrillary tangles and paired helical filaments in the final stages. These aggregates have a strong correlation with neuronal loss since they spread trans-synaptically throughout different parts of the brain. Therefore, while late-stage alterations cause irreversible neurodegeneration, early tau phosphorylation contributes to functional dysfunction (Arnsten et al. 2025).

### Neuroinflammation hypothesis

Recent research suggests that immunological dysregulation in the brain may be the principal cause of AD, hence increasingly underscoring the neuroinflammation theory. This theory posits that the onset and propagation of neuronal damage in Alzheimer’s disease are contingent upon persistent, dysregulated inflammation inside the central nervous system (CNS), orchestrated by innate immune cells such as microglia and astrocytes (Wong-Guerra et al. 2023a, b). Immunological surveillance, synaptic pruning, and the removal of waste, including misfolded proteins such as amyloid-β (Aβ), are all reliant on brain microglia. Astrocytes regulate neurotransmitters, maintain the blood-brain barrier, and nourish synapses. Alzheimer’s disease induces a phenotypic alteration in these glial cells that fosters persistent activation. Their production of proinflammatory cytokines, chemokines, reactive oxygen species, and nitric oxide harms neurons and induces synaptic dysfunction (Katsumoto et al. 2014). Amyloid plaques, functioning as a persistent stimulus, are thought to induce microglial activation in Alzheimer’s disease. NLRP3, a crucial component of the inflammasome, along with pattern recognition receptors (PRRs) such as TLRs and NLRs, identifies these plaques. The activation of the NLRP3 inflammasome produces IL-1β and IL-18, hence enhancing the local immune response. This response inhibits amyloid accumulation, resulting in neuronal injury and excessive inflammatory activity (Guo et al. 2015; Wang et al. 2023; Lu et al. 2024). Genetic variants associated with innate immunity, such as TREM2 (Triggering Receptor Expressed on Myeloid Cells 2), have been correlated with late-onset Alzheimer’s disease. TREM2 regulates microglial activation, phagocytosis, and survival. TREM2 mutations diminish microglial reactivity to amyloid plaques, hence hindering clearance and perhaps exacerbating plaque accumulation and neurotoxicity. Genome-wide association studies (GWAS) have identified numerous genes, including CD33 and CR1, that influence immunological responses and are associated with the risk of Alzheimer’s disease (Condello et al. 2017; Zhu et al. 2015). Neuroinflammation in Alzheimer’s disease extends beyond glial reactions and encompasses multiple levels. Inflammation can compromise the integrity of the blood-brain barrier (BBB), allowing inflammatory mediators and peripheral immune cells to infiltrate the brain, thereby exacerbating neuronal damage. Moreover, systematic inflammation brought on by infections or chronic diseases can increase CNS inflammation, suggesting a bidirectional interaction between peripheral and central immune reactions (Tran et al. 2022; Kiraly et al. 2023).

### Cholinergic hypothesis

A healthy brain requires acetylcholine to regulate arousal, memory, attention, and learning. The basal forebrain cholinergic system, particularly the nucleus basalis of Meynert, innervates the hippocampus and cerebral cortex, which are essential for higher-order cognitive functions. Numerous postmortem investigations on AD have identified a significant loss of cholinergic neurons in these regions. Brain damage results in a decrease in choline acetyltransferase (ChAT) activity, which synthesizes acetylcholine (ACh), hence lowering ACh levels (Haam and Yakel 2017). Although this concept was proposed several years ago, its significant impact on the development of contemporary therapeutic methods renders it still pertinent (MacHado et al. 2020; Chen et al. 2022a, b).

### Mitochondrial dysfunction and oxidative stress

Cellular energy failure and neurodegeneration are linked by mitochondrial dysfunction and oxidative stress, long-known causes of AD. By oxidative phosphorylation, mitochondria produce ATP, neurons’ main energy source. The brain’s high energy needs, especially in memory and cognition regions, make mitochondrial activity disruptions hazardous to neuronal survival and function (Misrani et al. 2021). In Alzheimer’s disease, mitochondrial dysfunction and oxidative stress reinforce each other. Increased reactive oxygen species (ROS) from malfunctioning mitochondria damage mtDNA, proteins, and membranes, limiting mitochondrial function. This loop worsens neuronal damage and accelerates the development of Alzheimer’s disease lesions, including neurofibrillary tangles and amyloid-β plaques. Greater Aβ levels may result from oxidative changes in enzymes that process amyloid precursor protein (APP), leading to greater breakage. Similarly, oxidative stress causes tau hyperphosphorylation and aggregation, forming neurofibrillary tangles (Zhu et al. 2013; D’Alessandro et al. 2025). In Alzheimer’s disease, aging, genetic mutations, and environmental contaminants cause mitochondrial malfunction and oxidative stress. The age-related reduction in mitophagy and other mitochondrial quality control processes makes it harder to remove damaged mitochondria, allowing them to multiply. Presenilin-1 mutations are linked to mitochondrial abnormalities and oxidative stress in familial Alzheimer’s disease. Environmental pollutants and lifestyle choices can worsen oxidative damage and mitochondrial deficiencies (Cai and Jeong 2020).

### Vascular contribution

In recent years, the complex link between cerebrovascular health and neurodegeneration has drawn attention to the function of vascular components in AD. Vascular contributions include anatomical and functional problems in cerebral blood vessels that may worsen or start Alzheimer’s disease. This viewpoint supports mixed dementia, where vascular pathology and conventional Alzheimer’s disease pathology coexist and interact, challenging the neurodegenerative view of the disease (Eisenmenger et al. 2022). Classical AD pathology and vascular impairment are interconnected and skewed. The presence of amyloid-β deposits in cerebral blood vessels can cause cerebral amyloid angiopathy (CAA), which can damage vascular integrity and increase the risk of microhemorrhages. Vascular amyloid can block blood flow and clearance channels, causing a feedback loop that increases parenchyma and artery amyloid deposition. Tau pathology is linked to vascular anomalies and ischemic neuronal susceptibility (Kuhn and Sharman 2023).

### Genetic factors

Our understanding of AD’s complicated etiology is shaped by inherited factors that affect risk, onset, and progression. Alzheimer’s disease can be familial (early-onset) or sporadic (late-onset), with genes playing different roles. Although rare, familial AD is caused by deterministic mutations following Mendelian inheritance patterns. Sporadic AD is caused by a complicated interaction of genetic risk variations and environmental factors (Sree et al. 2020; Ge et al. 2024; Andrews et al. 2023). APP, PSEN1, and PSEN2 are genes that cause early-onset familial Alzheimer’s disease (EOFAD). These genes directly process amyloid precursor protein, affecting amyloid-β peptide production and aggregation. Mutations in the APP or presenilin genes modify APP cleavage patterns, resulting in the amyloidogenic Aβ42 isoform, which forms toxic oligomers and fibrils. These mutations are highly penetrant, causing Alzheimer’s disease symptoms in people aged 30–50 (Hoogmartens et al. 2021). In contrast, most late-onset Alzheimer’s disease (LOAD) cases have a more complex genetic structure with many risk genes with different effects. The ε4 allele of the APOE gene is the primary genetic risk factor for late-onset Alzheimer’s disease (LOAD). Homozygous carriers at high risk for Alzheimer’s disease experience a dose-dependent acceleration in age of onset due to APOE ε4. APOE is critical for lipid metabolism, amyloid clearance, and neuroinflammation, linking metabolic and immunological pathways to Alzheimer’s disease. Along with APOE, GWAS have found loci involved in immunological regulation, endocytosis, lipid metabolism, and synaptic function that increase polygenic risk of Alzheimer’s disease (AD) (Andrade-Guerrero et al. 2023). Recent research emphasizes the role of rare immune response genetic variations, particularly TREM2, which affects microglial activation and neuroinflammation. TREM2 mutations increase Alzheimer’s disease risk, underscoring the role of neuroinflammatory pathways and innate immunity. Modern sequencing methods reveal new genetic variables and intricate gene-environment interactions that affect AD risk (Vogrinc et al. 2021).

### Metabolic and endocrine factors

Metabolic and endocrine dysfunctions are major causes of AD, demonstrating the complicated relationship between systemic physiological homeostasis and brain health. The brain’s complex dependence on metabolic equilibrium and hormone communication suggests that changes in these systems may affect neuronal function, synaptic stability, and cognitive function (Monte and Tong 2013). Impaired glucose metabolism is a hallmark of Alzheimer’s. The brain uses glucose for energy, but Alzheimer’s patients have reduced glucose absorption and use in the hippocampus and cortex. Hypometabolism before clinical symptoms suggests an initial disease episode. Brain insulin resistance, often known as “Type 3 diabetes,” affects insulin signalling pathways necessary for neuronal survival, synaptic plasticity, and amyloid clearance, linking metabolic dysfunction to Alzheimer’s disease (Monte 2012; Abdalla 2024). Thyroid, cortisol, and sex hormone imbalances can cause neurodegeneration and cognitive impairment. Subclinical thyroid malfunction and hypothyroidism may increase brain metabolism, plasticity, and Alzheimer’s risk. Cortisol increases hippocampus atrophy and cognitive decline due to HPA axis stress and instability. Reduced oestrogen and testosterone with age may explain postmenopausal women’s higher Alzheimer’s disease risk: decreased neuroprotection, amyloid buildup, and inflammation (Mooradian and Haas 2025; Kim et al. 2022). Lipid metabolism changes enhance Alzheimer’s risk. Neurons and synapses need cholesterol and other lipids for membrane fluidity and receptor function. Proper cholesterol transport and metabolism affect β-aggregation and amyloid precursor protein processing. APOE, a lipid transporter, links lipid metabolism to AD risk, specifically the λε4 variant that affects lipid balance and increases amyloid buildup (Liu et al. 2024; Yin 2022). Moreover, Mitochondrial metabolic disorders and endocrine illnesses may increase neuronal oxidative stress and energy depletion. Synaptic loss occurs owing to metabolic failure, protein misfolding, and neuroinflammation in AD (Ionescu-Tucker and Cotman 2021).

### Gut-Brain axis and microbiota

The gut-brain axis, which links the stomach and brain, is linked to Alzheimer’s. In this complex neurological, hormonal, and immunological framework, the gut microbiota, a diverse assemblage of intestinal bacteria, affects brain health and disease. Growing evidence links gut microbial dysbiosis to Alzheimer’s neurodegeneration (Kowalski and Mulak 2019). The blood-brain barrier, immune response, and metabolism are affected by intestinal microbiota. Systemic inflammation, intestinal permeability, and blood-brain barrier disruption result from dysbiosis. A “leaky gut” lets microbial metabolites, endotoxins such as lipopolysaccharides (LPS), and pro-inflammatory chemicals reach the brain, causing AD-related neuroinflammation. Chronic neuroinflammation from peripheral signals can cause cognitive decline by causing amyloid-β accumulation and tau hyperphosphorylation (Schoultz and Keita 2020). Moreover, brain development and neurotransmission are affected by gut microbiota’s modulation of SCFA, serotonin, and GABA production. Microorganisms can alter cognition, neurochemistry, and emotions. Gut microbiota affect cognitive function, since germ-free or antibiotic-treated animals behave differently (Ashique et al. 2024).

### Environmental and lifestyle factors

Environmental and lifestyle factors can affect brain resilience and neurodegeneration, making them important preventative and therapeutic targets. More environmental factors have been linked to Alzheimer’s. Chronic air pollution increases fine particulate matter and hazardous chemicals, causing brain neuroinflammation, oxidative stress, and amyloid pathology. Lead, mercury, and aluminium neurotoxicity research shows protein aggregation and mitochondrial dysfunction that may exacerbate dementia. Despite unknown causes, industrial pollution and pesticides threaten the environment (Park et al. 2024; Yegambaram et al. 2015). Lifestyle choices affect brain plasticity, metabolic regulation, and vascular health, affecting cognition and Alzheimer’s risk. Exercise improves cerebral blood flow, neurogenesis, and systemic inflammation, reducing cognitive decline and dementia. Conversely, inactivity accelerates cognitive aging (Ye et al. 2023a, b). Diet appears to affect Alzheimer’s risk. Anti-inflammatory antioxidants, polyphenols, and omega-3 fatty acids in Mediterranean diets may prevent Alzheimer’s disease. Conversely, processed meals, refined carbohydrates, and saturated fats can cause metabolic dysfunction, oxidative stress, and neurodegeneration (Picone et al. 2024). Lifestyle factors, including sleep quality, affect Alzheimer’s risk. Chronic sleep problems impair the brain’s glymphatic system, limiting the clearance of harmful chemicals like tau and amyloid-β. Sleep disorders increase amyloid buildup and neuroinflammation, indicating a reciprocal link with AD (Ragsdale et al. 2025). Mental health and stress affect sickness. Chronic psychological stress increases cortisol and neuroinflammation, which may worsen hippocampal damage and cognition. Depression in midlife or later life is a risk factor and prodromal symptom of Alzheimer’s (Knezevic et al. 2023).

### Prion-like spread of pathology

Prions-like pathogenic spread may explain Alzheimer’s spatiotemporal progression. Transmissible protein aggregation in prion illnesses may contribute to amyloid-β and tau misfolding in Alzheimer’s disease. The AD prion-like theory says brain-to-cell transmission, not transmissibility, accelerates disease progression (Colin et al. 2019; Walsh and Selkoe 2016). Protein misfolding, such as amyloid-β and hyperphosphorylated tau, can disrupt normally folded proteins. This templated misfolding spreads harmful oligomers and fibrils throughout brain circuitry. Cognitive decline is linked to entorhinal, hippocampal, and neocortex tau disease. Through exosomes, synaptic vesicles, or membrane penetration, neurons can discharge misfolded proteins into neighbouring cells. After entering recipient cells, pathogenic proteins misfold and interact with normal proteins. This spread may explain Alzheimer’s disease patients’ clinical neurodegeneration’s location and timing (Brettschneider et al. 2015; Aguzzi and Rajendran 2009).

## Drug treatment in alzheimer’s disease

Despite decades of research, developing effective AD treatments has been challenging. Many drugs have failed in clinical trials, while others have received controversial FDA approval with modest benefits. In this work, we will summarize all available drug therapies targeting AD (Abdallah 2024).

### Drugs targeting the amyloid cascade hypothesis

Since the Amyloid cascade hypothesis suggests that Aβ overproduction or impaired clearance leads to the formation of soluble Aβ oligomers and insoluble amyloid plaques, which trigger neurotoxicity, synaptic dysfunction, and inflammation, several pharmacological approaches have been explored to interfere with Aβ production, aggregation, or clearance, aiming to develop a successful treatment of AD (Wu et al. 2022).

#### Beta-Secretase (BACE)c inhibitors

Beta-Secretase-1 is the enzyme that cleaves amyloid precursor protein (APP) to initiate Aβ production. Thus, inhibiting BACE1 reduces Aβ levels. Many BACE1 inhibitor drugs have been developed. These drugs may be more effective in preclinical AD before significant neurodegeneration occurs. However, the off-target effects of BACE1 also process other proteins (e.g., neuregulin), leading to significant adverse effects.

*Verubecestat (Merck)* Failed in Phase III trials due to worsening cognition and side effects (weight loss, liver toxicity) (Egan et al. 2018).

*Lanabecestat (Eli Lilly/AstraZeneca)* Discontinued in Phase III for lack of efficacy (Zimmer et al. 2021).

*Atabecestat (Janssen)* Stopped in Phase II/III due to liver toxicity (Sperling et al. 2021).

#### Gamma-secretase modulators and inhibitors

Gamma-secretase cleaves APP to produce Aβ. Inhibiting it should reduce Aβ, but it also processes Notch signalling proteins, crucial for cell differentiation. Currently, Notch-sparing gamma-secretase modulators are being explored to selectively reduce Aβ42 without disrupting Notch signalling (Nordvall et al. 2023).

*Semagacestat (Eli Lilly)* stopped in Phase III due to increased worsening of cognition and skin cancer risk (Tagami et al. 2017).

*Avagacestat (Bristol-Myers Squibb)* Failed due to toxicity and lack of efficacy (Coric et al. 2015).

*Tarenflurbil (Flurizan; Myriad Genetics)* failed in Phase III clinical trials (Penninkilampi et al. 2016).

*Natural Gamma-Secretase modulators include* *SPI-014* which is isolated from *Actaea racemosa* extract and *2*,*3-Bis((Z)-4-methoxybenzylidene) succinonitrile* which is extracted from the marine fungus *Dichotomomyces cejpii*. Moreover, *Dihydroergocristine* is a natural agent that gained FDA approval for selective inhibition of amyloid precursor protein leading to significant Gamma-Secretase modulation (Abdallah 2024).

#### Immunotherapy (anti-amyloid monoclonal Antibodies)

These antibodies bind to Aβ and promote its clearance via the immune system. Passive immunotherapy (e.g., Aducanumab, Lecanemab) involves injecting antibodies, while active immunotherapy (e.g., AN1792, CAD106) stimulates the immune system to produce anti-Aβ antibodies.

*Aducanumab (Aduhelm; Biogen/Eisai)* It received accelerated FDA approval in 2021 as the first anti-amyloid therapy for early AD despite its controversial efficacy. Phase III trials showed mixed results, with some patients showing modest cognitive slowing but high rates of amyloid-related imaging abnormalities (Khanna et al. 2024). These results led to discontinued production in the U.S. and restricted real-world use. Aducanumab’s real-world limitations include high cost, narrow eligibility, uncertain cognitive benefits and significant amyloid-related imaging abnormalities risk; resulting in minimal real-world use outside research settings (Huang et al. 2025).

*Lecanemab (Leqembi, Biogen/Eisai)* It was FDA-approved in 2023 after showing a 27% slower decline in Phase III. However, amyloid-related imaging abnormalities remain a concern (Dyck et al. 2023). Long-term extension results from the open-label CLARITY-AD study showed that continuous lecanemab treatment over 3–4 years is associated with sustained slowing of cognitive and functional decline in early AD, with no major safety signals. This data suggests that early initiation and ongoing lecanemab therapy may delay progression from mild cognitive impairment due to AD to more advanced disease stages by several years, supporting long-term clinical benefit and consistent safety (Dyck et al. 2025).

*Donanemab (Eli Lilly)* it is currently in Phase III and showed 35% slowing of decline, but also high amyloid-related imaging abnormalities rates (Mintun et al. 2021). Recent TRAILBLAZER-ALZ & ALZ 2 phase 3 clinical trials showed that donanemab significantly slowed cognitive and functional decline in early symptomatic AD; however, almost half of the treated patients showed no clinical progression as compared to placebo. Benefits were greater in patients at earlier stages of disease, and the treatment led to substantial Aβ clearance (Dodel and Frölich 2025).

*AN1792 (Elan/Wyeth)* Discontinued in Phase II after 6% of patients developed meningoencephalitis (Vellas et al. 2009).

*CAD106 (Novartis)* Still in clinical trials, showing better safety but unclear efficacy (Vandenberghe et al. 2016).

However, in all these drugs, cognitive improvements are small, raising questions about real-world impact, especially noting that the huge cost of these treatments limits their patient accessibility (Song et al. 2022).

#### Aβ aggregation inhibitors

These drugs prevent Aβ from forming toxic oligomers and fibrils. Small-molecule inhibitors targeting specific Aβ oligomers are in preclinical development.

*Tramiprosate (Homotaurine, Neurochem Inc.)* Failed in Phase III due to lack of efficacy (Manzano et al. 2020).

*Scyllo-inositol (ELND005, AZD-103)* Several clinical trials have been initiated; however, none of them proved efficacy or cognitive benefit, so the drug was stopped in phase II (Ma et al. 2012).

Collectively, Aβ may be just one piece of the puzzle; tau-targeting drugs (e.g., anti-tau antibodies) and anti-neuroinflammatory drugs are now being explored. Combination Therapies targeting both Aβ and tau, or adding anti-inflammatory agents, could improve outcomes. Drugs enhancing Aβ clearance (via the glymphatic system) or targeting microglial activation are under investigation (Dias and Socodato 2025).

### Drugs targeting the tau hypothesis

In AD, tau undergoes abnormal hyperphosphorylation, misfolding, and aggregation, leading to the formation of NFTs, which disrupt intracellular transport, impair synaptic function, and correlate strongly with neuronal loss and cognitive decline. Because of this, tau has emerged as a key therapeutic target in AD. Several drug strategies are being investigated to modify tau pathology (Guo et al. 2022).

#### Tau aggregation inhibitors

These drugs aim to prevent tau molecules from clumping into toxic fibrils. A notable example is *LMTX* (a derivative of methylene blue), which has promising results in clinical trials (Congdon et al. 2023).

#### Enzymes regulating tau phosphorylation (PP2A activators and GSK-3β inhibitors)

Drugs that target the enzymes regulating tau phosphorylation are under investigation. Dysfunction of protein phosphatase 2 A (PP2A) and/or glycogen synthase kinase-3β (GSK-3β) contributes to tau hyperphosphorylation. These drugs show promising results in clinical trials.

*Memantine* and *Sodium selenate* are PP2A activators that are used for the inhibition of tau hyperphosphorylation (Johnson et al. 2024).

*Tideglusib and Lithium chloride* are examples of GSK-3β inhibitors that are currently being tested in the treatment of AD since they are used to stabilize tau in its normal state and prevent toxic modifications (Shri et al. 2023).

#### Microtubule stabilizers

These drugs aim to compensate for the loss of tau’s stabilizing function and small molecules that block tau-tau interactions. ***Methylthioninium chloride* (**Methylene blue) was the first microtubule stabilizer; however, it was ineffective in phase 2 clinical trials (Sarukhanyan and Dandekar 2023).

#### **Immunotherapy**

Anti-tau monoclonal antibodies are used to neutralize extracellular tau and limit its propagation between neurons. Antibodies such as *Zagotenemab*,* Tilavonemab*,* Semorinemab* and *Gosuranemab* have been tested in early-phase trials, with some showing favorable safety profiles, though large-scale efficacy results remain limited (Cai et al. 2025).

Although no tau-targeting drug has yet received regulatory approval, these strategies represent an important shift beyond amyloid-focused treatments. Current clinical trial outcomes suggest that tau-directed therapies may be most effective when initiated early, before extensive neurodegeneration has occurred. Furthermore, many researchers believe that a combination therapy approach, targeting both amyloid and tau, may be necessary to significantly alter the course of AD (Abdallah 2024).

### Drugs targeting the neuroinflammation hypothesis

The neuroinflammation hypothesis suggests that chronic activation of microglia and astrocytes in the brain leads to the release of pro-inflammatory cytokines, reactive oxygen species, and other toxic mediators that exacerbate neuronal damage. Consequently, modulation of neuroinflammation has emerged as a promising therapeutic strategy (Mostafa and Asaad 2023).

Several classes of drugs have been investigated to target neuroinflammation in AD.

#### Nonsteroidal anti-inflammatory drugs (NSAIDs)

 Were among the first to be studied, based on epidemiological evidence suggesting that long-term NSAID use reduced AD risk. Agents such as *ibuprofen*, *naproxen*, *sulindac sulfide*, and *indomethacin* were evaluated in clinical trials; however, most failed to show significant benefits when given to patients with established AD due to their poor brain tissue penetration. These results suggest that NSAIDs may be more effective as preventive rather than therapeutic agents (Kaduševičius 2021).

NSAID-derived carboxylic acid derivatives (e.g., *Tarenflurbil*) were proposed as therapeutic candidates for the treatment of AD; however, a phase III clinical study failed due to their limited efficacy and inadequate brain penetration (Athar et al. 2021).

*JNJ-40,418,677* (Janssen) is an analog of Flurbiprofen that has sufficient lipophilicity to penetrate the brain; however, its safety profile still needs extensive evaluation (Rombouts et al. 2021).

*EVP-0015962* (Forum Pharmaceuticals) is a Tarenflurbil analog whose high lipophilicity caused discontinuation of its clinical trials after phase II (Rajanna et al. 2025).

*Itanapraced* (Chiesi Pharmaceuticals) was found to reduce microglial activation.

BIIB042 (Biogen) showed considerable potency and decreased the amyloid plaque burden; however, it exhibited poor brain penetration (Zhang et al. 2022).

#### Microglial modulators

Were designed to control microglial overactivation without suppressing their protective functions. Drugs targeting triggering receptor expressed on myeloid cells 2 (TREM2) receptors are being developed, as TREM2 plays a critical role in regulating microglial responses to amyloid plaques. Experimental therapies enhancing TREM2 signaling aim to shift microglia from a pro-inflammatory state to a more phagocytic, plaque-clearing phenotype (Zhang et al. 2022).

#### Cytokine inhibitors and immune modulators

Have also been proposed. Drugs targeting tumor necrosis factor-alpha (TNF-α), interleukin-1β (IL-1β), and other inflammatory mediators are under exploration. For example, TNF-α inhibitors like ***etanercept***, widely used in autoimmune conditions, are being investigated for their ability to reduce neuroinflammatory cascades in AD. Additionally, modulators of the NLRP3 inflammasome, a key driver of inflammatory activation in microglia, have shown promising results in preclinical models (Ogunmokun et al. 2021).

#### Other experimental drugs include *colchicine*

Traditionally used for gout, which acts as a broad anti-inflammatory by disrupting microtubule assembly and limiting cytokine release (Ogunro et al. 2025). Furthermore, natural compounds with anti-inflammatory properties, such as *curcumin* and *resveratrol*, are being explored as adjunct therapies due to their neuroprotective effects (Abdul-Rahman et al. 2024; Puranik et al. 2025).

Despite the intense interest, clinical trials of anti-inflammatory therapies in AD have so far yielded mixed outcomes. This may be due to the complexity of neuroinflammation, which involves both protective and harmful processes, as well as the fact that interventions may need to begin very early in the disease process. Nevertheless, targeting neuroinflammation remains an important therapeutic approach. While no anti-inflammatory agent has yet been approved specifically for AD, ongoing trials continue to refine these approaches (Wong-Guerra et al. 2023a, b).

### Drugs targeting the cholinergic hypothesis

The cholinergic hypothesis is one of the earliest and most widely studied theories in the pathophysiology of AD. It proposes that the progressive cognitive decline observed in AD is strongly associated with a deficit in the neurotransmitter ACh. Based on this, therapeutic strategies have been developed to enhance cholinergic transmission, aiming to temporarily improve or stabilize cognitive function in AD patients (Chen et al. 2022a, b).

#### Acetylcholinesterase inhibitors (AChEIs)

The main class of drugs used to target this pathway is acetylcholinesterase inhibitors (AChEIs). These agents inhibit the enzyme AChE, which normally breaks down ACh in the synaptic cleft. By blocking this enzyme, AChEIs increase the concentration and duration of action of ACh at the synapse, thereby compensating for the cholinergic deficit. Commonly used AChEIs include:

*Donepezil* is a selective reversible AChEI with once-daily dosing and relatively favorable tolerability that can be used for severe stages of AD (Pardo-Moreno et al. 2022).

*Rivastigmine* inhibits both AChE and butyrylcholinesterase and can be administered orally or via a transdermal patch to reduce the gastrointestinal side effects. It is approved for the management of mild to moderate AD (Nguyen et al. 2021).

*Tacrine* also inhibits both AChE and butyrylcholinesterase but was withdrawn from the market due to its significant hepatotoxicity (Sundius and Brandán 2023).

*Galantamine* is an AChEI and also acts as an allosteric modulator of nicotinic ACh receptors, enhancing their response to endogenous ACh, which may provide added cognitive benefits. Despite these therapeutic effects, side effects such as nausea, vomiting, diarrhea, loss of appetite, and bradycardia are common and can limit tolerability. Galantamine is approved for the management of mild to moderate AD (Baakman et al. 2022).

Clinical trials have shown that these drugs can lead to modest improvements in memory, attention, and global cognitive performance, and may help with daily functioning. However, the effects are symptomatic rather than disease-modifying, and benefits typically last for months to a few years before disease progression continues. Currently, AChEIs remain the cornerstone of cholinergic-based therapy.

Recent evidence shows that cholinesterase inhibitors provide only modest symptomatic benefit beyond short-term use, with most randomized trials lasting ≤ 6 months and limited robust long-term (> 1–2 years) placebo-controlled efficacy data, making sustained cognitive slowing uncertain. Long-term observational studies suggest small, persistent effects on cognition and mortality; however, overall benefits are modest and variably sustained, with inconsistent evidence on clinically meaningful slowing of decline over time (Zuliani et al. 2024).

#### Nicotinic and muscarinic receptor agonists

Beyond AChEIs, other strategies have been explored. One example is the use of selective cholinergic receptor agonists, which directly stimulate muscarinic or nicotinic receptors. Since nicotinic receptors are important for memory, learning, and cognitive processes, drugs that target these rather than all cholinergic receptors have been developed.

These drugs are comparatively safer than AChEIs (Verma et al. 2018).

*ABT-126* is a selective nicotinic receptor agonist developed as a monotherapy for mild to moderate AD. ABT-126 was usually well tolerated and showed some improvement in cognition in people with mild to moderate AD in phase 2a clinical studies (Haig et al. 2016).

*Oxotremorine* is a selective muscarinic receptor agonist developed for the treatment of AD. Its use showed controversial results (Nuzzo et al. 2023).

*Xanomeline* is a selective muscarinic agonist that showed some promise in clinical trials, but was associated with intolerable adverse effects (McIntyre 2025).

#### Ach precursors

Efforts to develop ACh precursors (such as *lecithin* or *choline supplementation*) have not shown consistent clinical benefit (Amenta and Tayebati 2008).

In summary, drugs targeting the cholinergic hypothesis play an important role in the management of AD, providing symptomatic relief and temporary stabilization of cognitive decline. While they do not halt or reverse the underlying neurodegeneration, they remain a mainstay in clinical practice due to their modest but meaningful benefits in quality of life and daily functioning (Chen et al. 2022a, b).

### Drugs targeting the mitochondrial dysfunction and oxidative stress hypothesis

In AD, evidence shows mitochondrial abnormalities, including reduced activity of key respiratory chain enzymes, altered mitochondrial dynamics, and increased susceptibility to oxidative damage. These defects lead to excessive oxidative stress, lipid peroxidation, protein oxidation, and DNA damage, all of which contribute to neuronal injury and accelerate disease progression (Bhatia et al. 2022).

#### Antioxidants

Therapeutic strategies have focused on antioxidants and mitochondrial protective agents that aim to reduce oxidative stress, stabilize mitochondrial function, and enhance neuronal survival.

*vitamin E (α-tocopherol)* is a lipid-soluble antioxidant that scavenges free radicals and reduces lipid peroxidation in neuronal membranes and is widely studied in AD. High-dose vitamin E supplementation can modestly slow functional decline in patients with mild to moderate AD, although cognitive improvements are limited. However, concerns remain regarding the safety of high doses, which may increase the risk of hemorrhagic stroke or mortality in some populations (Pelczarski et al. 2025).

*Caffeine* is an antioxidant that inhibits amyloid plaque production in AD (Yelanchezian et al. 2022).

*Selegiline* is a monoamine oxidase-B inhibitor with antioxidant properties. Selegiline has been tested in AD and shown slight benefits in cognition and daily functioning, mainly via its antioxidant properties (Basir et al. 2025).

*Silibinin*, a herbal flavonoid antioxidant derived from *Silybum marianum*, prevents memory impairment and shows promising results in the treatment of AD (Liu et al. 2023).

*Curcumin* is a natural polyphenol with antioxidant, anti-neuroinflammatory and anti-amyloid properties. Recent studies suggest that it acts as an AChEI (Abdul-Rahman et al. 2024).

Q*uercetin* is a polyphenolic compound showing neuroprotective properties as it reduces β-amyloidosis, tau pathology, and inhibits astrocyte and microglia activation (Kaur et al. 2024).

*Luteolin*, a flavonoid antioxidant with neuroprotective properties, shows promising results in the treatment of AD (Delgado et al. 2021).

*Melatonin* possesses strong antioxidant properties, thus reducing tau hyperphosphorylation and improving symptoms of AD as memory and cognition (Steinbach and Denburg 2024).

*Ginkgo biloba extract (EGb 761)* is a herbal preparation with antioxidant and neuroprotective properties. Several trials have evaluated its efficacy in dementia. It showed modest improvements in cognitive and behavioral symptoms. It is currently used as a complementary therapy in some regions (Xie et al. 2022).

#### Mitochondria-targeted antioxidants

*MitoQ* (mitoquinone) and *SkQ1* were designed to selectively accumulate within mitochondria and neutralize ROS at their source. Preclinical studies suggest they may protect against mitochondrial dysfunction and amyloid-induced toxicity (Shinn and Lagalwar 2021).

*Coenzyme Q10* (ubiquinone) and its analogs, such as *Idebenone*, have also been investigated. Coenzyme Q10 is a vital component of the electron transport chain and acts as an endogenous antioxidant. Supplementation aims to restore mitochondrial energy production and reduce oxidative damage. While preclinical models showed promising results, clinical trials in AD have not demonstrated robust benefits. Idebenone similarly showed limited efficacy in human studies (Sheykhhasan et al. 2022).

*lipoic acid*, *vitamin C* and *glutathione* are antioxidants that can regenerate other antioxidants. Although widely studied, issues with bioavailability and inconsistent clinical outcomes have prevented their widespread adoption as standard treatments of AD (Khan et al. 2022).

In summary, drugs targeting mitochondrial dysfunction and oxidative stress in AD aim to counteract one of the key mechanisms of neurodegeneration. While agents such as vitamin E, selegiline, and Ginkgo biloba have shown modest benefits, no antioxidant or mitochondrial stabilizer has yet demonstrated strong disease-modifying effects in large clinical trials (Bhatia et al. 2022). However, the use of antioxidants in AD shows limited and inconsistent clinical benefit, with most trials failing to demonstrate meaningful slowing of cognitive decline. Key limitations include poor brain bioavailability, late intervention timing, oversimplification of oxidative stress in such a multifactorial disease, and mixed or negative trial outcomes (Zuliani et al. 2024).

### Glutamatergic drugs

The glutamatergic system is the major excitatory neurotransmitter pathway in the CNS. Glutamate plays a critical role in learning and memory through the activation of glutamate receptors, especially NMDA receptors. However, in AD, there is evidence of glutamatergic dysfunction, where excessive and chronic activation of NMDA receptors leads to excitotoxicity. This excitotoxicity contributes to the progressive neurodegeneration and cognitive decline seen in AD (Babaei 2021).

*Memantine*, an uncompetitive NMDA receptor antagonist, is the most widely used and clinically approved drug in moderate to severe AD. Clinical trials of memantine have shown modest benefits in cognition, daily functioning, and global clinical status. In moderate to severe stages of AD, patients taking memantine often demonstrate a slower decline in memory, attention, language, and ability to perform daily activities compared to placebo. While the effects are not curative or disease-modifying, they provide meaningful symptomatic relief and improve quality of life for both patients and caregivers.

Memantine is often used as monotherapy in moderate to severe AD, but it is also frequently prescribed in combination therapy with AChEIs. This dual approach targets both the cholinergic and glutamatergic systems, aiming to maximize cognitive and functional benefits.

Side effects of memantine are generally mild compared to cholinesterase inhibitors. The most common adverse effects include dizziness, headache, confusion, constipation, and occasionally hallucinations. These are usually dose-dependent and often improve with continued treatment. Memantine is generally well tolerated, making it a useful option for elderly patients who may not tolerate AChEIs well due to gastrointestinal or cardiac side effects (Tang et al. 2023).

Other investigational glutamatergic agents have been studied, including novel NMDA & AMPA receptor modulators and metabotropic glutamate receptor (mGluR) agents; however, most have not advanced beyond clinical trials due to limited efficacy or safety concerns (Babaei 2021).

### Phosphodiesterase inhibitors (PDEIs)

Phosphodiesterase inhibitors (PDEIs) are a class of drugs that regulate intracellular signaling by preventing the breakdown of cyclic nucleotides such as cyclic adenosine monophosphate (cAMP) and cyclic guanosine monophosphate (cGMP). These messengers play a crucial role in neuronal signaling, synaptic plasticity, learning, and memory. Since AD is characterized by synaptic dysfunction, impaired plasticity, and neuronal loss, PDE inhibition has been proposed as a therapeutic strategy to restore normal cyclic nucleotide signaling and enhance cognition. It has also been proposed that PDEIs reduce amyloid-β production, tau phosphorylation, and neuroinflammation, making them multi-target agents in AD therapy (Chandra et al. 2024).

Classes of PDEIs include:

#### PDE1 inhibitors

Regulate both cAMP and cGMP signaling and are highly expressed in the hippocampus, cortex, and striatum. Through PDE1 inhibition, intracellular cyclic nucleotide levels rise, leading to improved neuronal signaling and neuroprotection.

*Vinprocetine* is a PDE1 inhibitor with vasodilatory and neuroprotective properties that has been used in some countries for cerebrovascular disorders. Preclinical studies suggest it improves memory, increases cerebral blood flow, and reduces oxidative stress (Shekarian et al. 2023).

#### PDE3 inhibitors

Primarily hydrolyze cAMP, and are involved in neuronal survival and plasticity. PDE3 inhibition increases cAMP, which supports memory consolidation.

*Cilostazol* is an antiplatelet and vasodilator drug used for stroke prevention that has shown promise in mild cognitive impairment in AD via improving cerebral blood flow and reducing amyloid pathology in preclinical studies. Clinical pilot studies in humans suggest cilostazol may slow cognitive decline when added to standard AD therapy (Saito et al. 2023).

#### PDE4 inhibitors are expressed in neurons and glial cells, regulating cAMP levels

*Roflumilast* is a drug approved for chronic obstructive pulmonary disease that was found to enhance synaptic plasticity, improve memory in animal AD models, and reduce neuroinflammation. However, gastrointestinal side effects limit its tolerability (Possemis et al. 2024).

*Zatolmilast* use showed improvement in memory and cognitive functions. Clinical trials reported that its use is safe and well-tolerated with fewer gastrointestinal side effects.

#### PDE5 inhibitors regulate cGMP degradation in vascular and neural tissue (Zhao et al. 2024)

*Sildenafil*, *Tadalafil* and *Vardenafil* *were found to im*prove cerebral blood flow, enhance synaptic transmission, and promote neurogenesis. Preclinical studies have shown a reduction in amyloid burden and an improvement in cognition. Ongoing clinical trials are testing the cognitive benefits of these compounds in AD (Abouelmagd et al. 2024).

#### PDE9 inhibitors hydrolyze cGMP and are highly expressed in the hippocampus and cortex

*PF-04447943* and *BAY 73-6691* are PDE9 Inhibitors that show improvement in learning and memory in AD models. However, clinical trials have shown limited efficacy (AbdAlla et al. 2021).

#### Dual PDE inhibitors

Are under development to achieve broader enhancement of cyclic nucleotide signaling while potentially lowering side effects by using lower doses of each component.

*Propentofylline* is a broad-spectrum PDEI that shows improvement in cognition and daily life activities in phase III mild-to-moderate AD (AbdAlla et al. 2021).

In summary, PDEIs represent a promising therapeutic class for AD by targeting impaired cyclic nucleotide signaling, synaptic dysfunction, and neuroinflammation. However, challenges with safety, tolerability, and mixed clinical results highlight the need for further research (Chandra et al. 2024).

### α-Secretase activators

α-Secretase activators represent a promising therapeutic approach in AD by promoting the non-amyloidogenic processing of amyloid precursor protein (APP). Activation of α-secretase, particularly ADAM (A Disintegrin and Metalloprotease)10 and ADAM17 enzymes, leads to the cleavage of APP within the Aβ domain, thereby reducing Aβ accumulation and producing soluble APPα, which has neuroprotective and synaptogenic effects.

*Retinoic acid*, *Melatonin* and *Etazolate (EHT-0202)* are potential α-secretase activators, where they enhance soluble APPα formation. Clinical trials show promising cognitive.

improvements with good tolerability in patients with mild-to-moderate AD (Kumar and Bansal 2022).

### Glutaminyl cyclase inhibitors

These agents represent a novel disease-modifying therapeutic approach in the treatment of AD by targeting the enzymatic activity responsible for the formation of neurotoxic pyroglutamate-modified amyloid-β (pE-Aβ) peptides. These truncated and cyclized Aβ species are highly aggregation-prone, more resistant to degradation, and exhibit enhanced neurotoxicity compared to full-length Aβ, thereby contributing significantly to amyloid plaque formation and AD progression.

*PQ912* (*Varoglutamstat*) is a potential glutaminyl cyclase inhibitor that blocks the generation of pE-Aβ, thereby reducing amyloid burden, synaptic dysfunction, and cognitive decline. Early clinical studies with PQ912 have demonstrated target engagement and promising effects on synaptic function (Chen et al. 2023).

### Drugs acting on 5-HT receptors

Drugs targeting serotonin (5-HT) receptors have gained attention in AD due to the critical role of serotonergic signaling in cognition, mood, and neuroprotection. Several 5-HT receptor subtypes, including 5-HT₄, 5-HT₆, and 5-HT₇, are expressed in brain regions affected by AD.

*Prucalopride* and *Velusetrag* are 5-HT₄ receptor agonists that show promise in enhancing the release of ACh, promoting neurogenesis, and increasing α-secretase activity, thereby offering both symptomatic and disease-modifying potential (Patel et al. 2023).

*Idalopirdine* and *Intepirdine* are 5-HT₆ receptor antagonists that improve memory and executive functions by modulating neurotransmitter release (Lang et al. 2021).

While clinical outcomes have been mixed, 5-HT receptor-acting drugs remain an important area of AD research for both symptomatic relief and modulation of disease progression.

### Drugs acting on GABA receptors

#### GABA-A receptor agonists

These agents emerged as potential therapeutic drugs in the treatment of AD due to their ability to restore inhibitory neurotransmission in the brain. Early stages of AD are associated with neuronal hyperexcitability and network dysfunction, particularly in the hippocampus and cortex, which can contribute to cognitive impairment and increase the risk of seizures. Moreover, GABA-A receptor agonists regulate sleep patterns and reduce behavioral disturbances, which are common in AD patients (Ali et al. 2023).

*Muscimol* is a direct GABA-A receptor agonist that stabilizes neural circuits and potentially improves cognitive function (Rivera-Illanes and Recabarren-Gajardo 2024).

*Etazolate* (*EHT0202*) is a GABA-A receptor agonist that shows anti-neuroinflammatory effects accompanied by cognitive improvement in AD patients (Abdul Manap et al. 2024).

*Propofol* is another GABA-A receptor agonist used for reducing Aβ aggregation and improving cognitive function in AD (Eryilmaz et al. 2024).

#### GABA-B receptor antagonists

These agents are being investigated as potential cognitive enhancers in AD due to their ability to modulate inhibitory neurotransmission and promote excitatory signaling. Since excessive GABA-B receptor activity may suppress the release of key neurotransmitters such as glutamate and ACh.

*CGP-35,348* and *SGS742* (*CGP36742*) are GABA-B receptor antagonists that have demonstrated improvements in cognitive performance in preclinical models and early human clinical trials (Vlachou 2022).

Unlike GABA-A receptor activation, which directly affects neuronal excitability, GABA-B receptor antagonism works through presynaptic mechanisms, offering a subtler way to rebalance neurotransmission without the sedative risks associated with GABA-A receptor agonists. Further clinical studies are needed to confirm efficacy and safety in AD (Carello-Collar et al. 2023).

### Drugs targeting the vascular contribution hypothesis

Cerebral hypoperfusion, microvascular damage, and impaired clearance of Aβ through the BBB contribute to the initiation and progression of cognitive decline seen in AD pathology. Vascular risk factors such as hypertension, diabetes, dyslipidemia, and stroke are strongly associated with dementia; therefore, therapies aimed at improving vascular health and cerebral blood flow are increasingly investigated for AD management (Scheffer et al. 2021).

### Antihypertensive agents

Since hypertension accelerates small vessel disease and contributes to impaired Aβ clearance, clinical studies have shown that antihypertensive drugs such as angiotensin-converting enzyme inhibitors (e.g., *perindopril*), angiotensin receptor blockers (e.g., *losartan*) and calcium channel blockers (e.g., *nimodipine*) may not only lower blood pressure but also exert neuroprotective effects. These drugs improve endothelial function, enhance cerebral perfusion and reduce oxidative stress; thus, may reduce AD risk and slow cognitive decline (Law and Yeong 2021).

### Statins

Statins lower cholesterol and reduce vascular risk, but they may also decrease Aβ production by modulating cholesterol-dependent amyloidogenic processing. Drugs like ***atorvastatin*** and ***simvastatin*** have been evaluated in clinical trials, where some evidence suggests that they may reduce the risk of developing dementia (Olmastroni et al. 2022).

### Antiplatelets and anticoagulants

Since cerebrovascular dysfunction often involves microthrombi and impaired perfusion, antiplatelet drugs such as *aspirin* and c*lopidogrel* along with novel oral anticoagulants have been proposed to improve cerebral blood flow. However, clinical data remain inconclusive, and concerns about bleeding risks limit widespread adoption (Toribio-Fernandez et al. 2024; Lee et al. 2023).

### Anti-diabetic drugs

Since diabetes is a major vascular risk factor for AD, drugs like *metformin* and GLP-1 receptor agonists (e.g., *liraglutide*) have been evaluated for their dual role in metabolic and vascular regulation. These agents may improve endothelial function and cerebral blood flow (Goodarzi et al. 2023).

### Lifestyle and multimodal vascular interventions

Multimodal vascular risk management, combining antihypertensives, statins, antiplatelets, and lifestyle modifications, has shown effectiveness in slowing cognitive decline. The FINGER trial (Finnish Geriatric Intervention Study) highlighted the benefit of managing vascular and metabolic risk factors together (Barbera et al. 2023).

### Drugs targeting the gut–brain axis and microbiota

Since growing evidence suggests that gut microbiota dysbiosis influences neuroinflammation, Aβ aggregation, tau hyperphosphorylation, and BBB integrity. Mechanistically, bacterial metabolites (short-chain fatty acids, lipopolysaccharides) and immune modulation can either promote or attenuate neurodegeneration. Therefore, interventions aimed at modifying gut microbiota are increasingly being studied as potential therapeutic strategies for AD (Wiatrak et al. 2022).

### Probiotics and prebiotics

Probiotics are live beneficial microorganisms that restore a healthy gut microbiome, while prebiotics are dietary fibers that stimulate beneficial bacterial growth. Clinical and preclinical studies have shown that probiotic strains such as Lactobacillus and Bifidobacterium can improve cognitive performance, reduce oxidative stress, and attenuate neuroinflammation. Prebiotics like inulin and fructo-oligosaccharides enhance the production of short-chain fatty acids, which may protect neurons and improve synaptic plasticity. Combination therapy (synbiotics) has also demonstrated beneficial effects in experimental AD models (Thakkar et al. 2023).

### Fecal microbiota transplantation (FMT)

FMT involves transferring gut microbiota from healthy donors to restore intestinal balance in patients with dysbiosis. Animal studies suggest that FMT can reduce amyloid deposition and improve memory functions. Early clinical research in humans is limited but promising, with case reports showing cognitive improvement. Safety, donor selection, and standardization remain important considerations for the routine use of AD (Park et al. 2021).

### Antibiotics and microbiota-modulating drugs

Antibiotics can reshape gut microbiota composition, thereby indirectly modulating neuroinflammatory responses. For instance, rifaximin, a non-absorbable antibiotic, has been investigated for its ability to alter gut microbiota and reduce systemic inflammation in AD patients. However, long-term use of broad-spectrum antibiotics may cause harmful dysbiosis, limiting their application. Future therapies may involve narrow-spectrum or targeted microbiota-modulating compounds (Fulop et al. 2021).

#### Dietary modification

Certain dietary compounds act as microbiota modulators and exert neuroprotective effects. The Mediterranean diet and foods rich in polyphenols (e.g., resveratrol, curcumin) have been associated with improved cognition and gut health. Omega-3 fatty acids and specific vitamins (B-complex, vitamin D) also influence gut microbiota composition, which may help reduce AD progression (Xue et al. 2022).

**Fig. 1:**
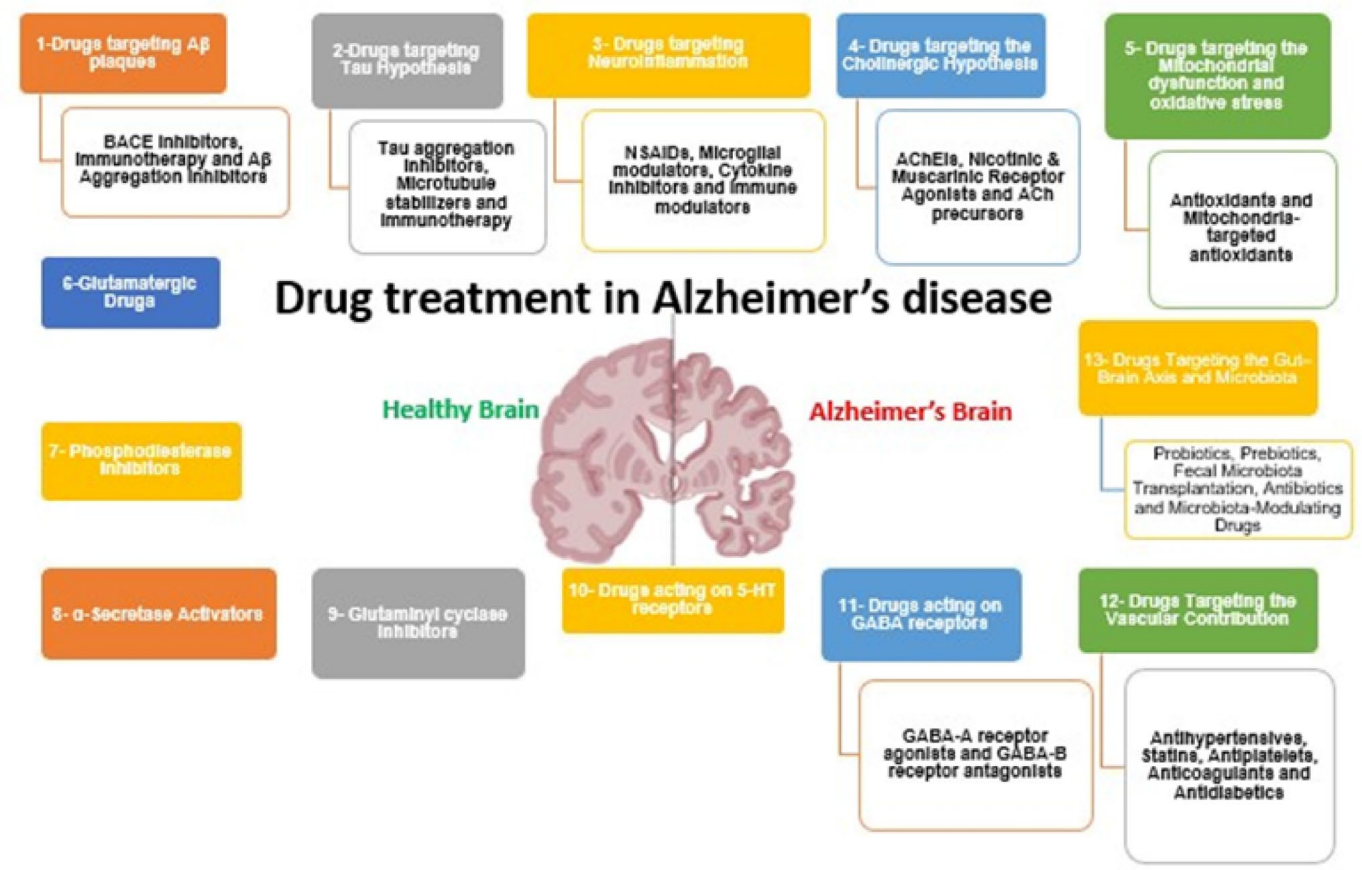
Overview of current and emerging drug treatment strategies in Alzheimer’s disease

## Gaps in alzheimer’s disease treatment

**Fig. 2:**
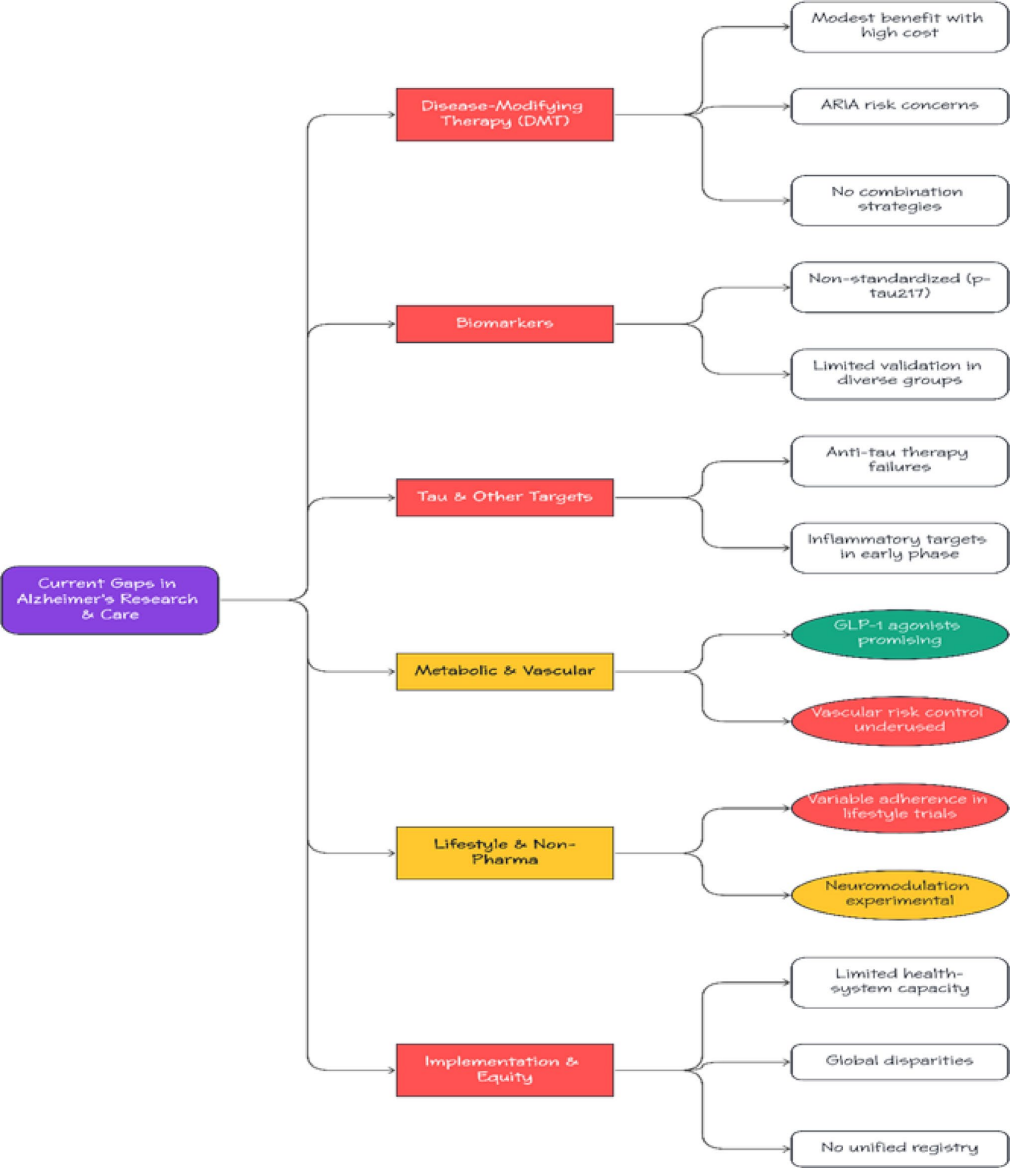
Major gaps and unmet needs in Alzheimer’s disease treatment and research

Despite major advances in Alzheimer’s disease research, scientific, clinical, and societal inequities impede diagnosis, treatment, and patient care. Deficits in diagnosis, treatment, customized care, monitoring, scientific understanding, and societal acceptance of Alzheimer’s disease hamper development in all these categories. These gaps reveal where policy, financing, and innovation should focus. Closing these gaps through multidisciplinary teamwork, technological integration, individualized methods, and public awareness can make Alzheimer’s disease controllable, preventable, and treatable. Figure 2 integrates the current knowledge, identifies unmet needs, and highlights future research directions in AD.

### Gap in early and accurate diagnosis

Alzheimer’s disease is challenging to identify as neurodegenerative processes commence several years or even decades prior to the manifestation of symptoms. Gold standard indicators, including amyloid PET imaging and cerebrospinal fluid testing, are invasive, expensive, and unsuitable for clinical use. Blood-derived biomarkers and computerized cognitive assessments are compelling but remain under examination. Consequently, several individuals are misdiagnosed or diagnosed late with dementias or mental diseases after experiencing significant cognitive decline and neuronal deterioration. The signs of Alzheimer’s disease overlap with normal aging and other neurological disorders, complicating diagnosis. Diagnostic limitations impeded focused therapy and obstructed care coordination (Gunes et al. 2022; Varesi et al. 2022; Zeng et al. 2024).

### Gap in effective, disease-modifying treatments

Authorized Alzheimer’s drug treatments only treat cognitive and behavioral symptoms, not neurodegeneration. While memantine and cholinesterase inhibitors help certain patients, they have little effect on pathogenesis. Despite debate regarding their therapeutic potential, aducanumab and lecanemab lower amyloid plaque load (Wojtunik-Kulesza et al. 2023). Alzheimer’s disease is multifaceted, with amyloid and tau aggregation, neuroinflammation, vascular dysfunction, oxidative stress, and synaptic loss, therefore, single-pathogenic target treatments often fail. These issues are compounded by clinical test variability, poor patient selection indicators, and difficulty detecting moderate cognitive changes. A reliable Alzheimer’s treatment is still lacking (Brockmann et al. 2023).

### Gap in personalized and precision medicine

The “one-size-fits-all” Alzheimer’s disease treatment ignores genetic, genomic, and clinical heterogeneity (Bieber et al. 2017). APOE genotype affects risk and progression, but clinical practice and treatment procedures have not included this knowledge (Kane 2024). Clinical trials may not stratify by biomarkers or genetic profiles, making it difficult to identify patient subgroups that may benefit most from therapies (Abdelnour et al. 2022). Genetic and molecular profiles are underutilized, making it harder to customize treatments to specific patients and hide the complex biological factors that may underlie therapeutic success. Thus, individualized and targeted Alzheimer’s disease treatments based on each patient’s biology are still unattainable (Arafah et al. 2023).

### Gap in non-pharmacological and supportive care

Complete Alzheimer’s disease treatment needs caregiver assistance, equal healthcare access, lifestyle and psychological therapies. Cognitive training, physical exercise, dietary adjustments, and social involvement prevent cognitive decline, but they are rarely employed clinically (Vidyanti et al. 2025). Family caregivers lack expertise, tools, and respite programs, creating stress and lower quality of life for themselves and patients (Lindeza et al. 2024). Low-income and rural residents have trouble accessing memory care facilities. Racial and ethnic minority communities, who are underrepresented in clinical trials and have problems accessing diagnosis and treatment, have extra obstacles (Wiese et al. 2023). Fixing these difficulties is essential for holistic, patient-centered Alzheimer’s care.

### Gap in continuous monitoring and real-world data

The management of Alzheimer’s disease is deficient in the absence of real-time cognitive and functional monitoring tools. Traditional clinic encounters capture a patient’s condition only during specific, episodic interactions, overlooking subtle or transient changes that could signify deterioration or therapeutic efficacy. Clinical practice and research inadequately utilize digital health technologies such as wearable sensors, smartphone applications, and in-home monitoring systems. The integration and interpretation of digital data complicate the formulation of personalized treatment strategies. By adopting a patient-centered approach, these obstacles can be eliminated, allowing for the implementation of continuous monitoring to facilitate early and more intensive treatment of Alzheimer’s disease (Chinner et al. 2018; Marvasti et al. 2024).

### Gap in understanding and targeting disease mechanisms

The complete pathophysiology, encompassing causative processes and illness development, remains inadequately understood. While Amyloid biomarkers are widely recognized, the development and validation of biomarkers for tau pathology, neuroinflammation, and synaptic dysfunction are challenging, hindering disease characterisation. The challenge of transferring preclinical findings into clinical applications is evidenced by the ineffectiveness of numerous therapeutic targets un human clinical trials. A deeper understanding the molecular and cellular mechanisms of Alzheimer’s disease is crucial for identifying novel therapeutic targets and formulating more efficacious, mechanism-based treatments (Monteiro et al. 2023).

### Gap in public awareness and stigma reduction

Alzheimer’s treatment is impeded by social factors. Early intervention and quality of life are impeded by prejudices that depict dementia as irreversible. The public is unable to identify early Alzheimer’s symptoms or modifiable risk factors. Consequently, the likelihood of a diagnosis decreases, and the options for risk reduction and prevention are restricted. In research and policy, patient and caregiver perspectives may be misinterpreted. A discrepancy exists between scientific progress and actuality. These cultural barriers must be overcome in order to deliver person-centered medical, social, and affective Alzheimer’s care (Rasmussen and Langerman 2019; Porsteinsson et al. 2021).

## Future perspective

### Amyloid research is becoming realistic

The FDA’s approval of lecanemab (Leqembi) in July 2023 and donanemab (Kisunla) in July 2024 has shifted the focus from “if” to “how” to implement disease-modifying therapy, emphasizing patient selection, ARIA risk management, monitoring, and healthcare system (Li et al. 2024; Palmqvist et al. 2025).

ARIB (amyloid-related imaging abnormalities) will impact future protocols, with APOE ε4 carriers and expedited plaque removal posing increased risk. Genotype-informed consent and MRI procedures are recommended, and 2024–2025 guidance and registries aim to standardize safety and outcomes monitoring (Ono and Tsuji 2020; Amin and Harris 2021).

Strategically, trials like TRAILBLAZER-ALZ 2 support “treat-to-target with stop rules” after amyloid clearance, which might be applied to earlier disease stages and combination treatment (Hodsdon et al. 2025).

### Faster and simpler diagnosis: blood biomarkers are becoming clinically available

Numerous studies from 2023 to 2025 showed accuracy comparable to CSF and PET standards for plasma phosphorylated-tau, particularly p-tau217, which will revolutionize case identification, triage to PET/CSF, and eligibility assessment for disease-modifying therapy. Recently, plasma p-tau231 has emerged as a prominent biomarker, exhibiting great sensitivity for early amyloid pathology and indicating potential utility in preclinical and prodromal stages of Alzheimer’s disease. Specialist care use suggestions are preliminary in the Alzheimer’s Association’s 2025 practice advice, with complete implementation guidance under development (Jack et al. 2024). Updated diagnostic frameworks for 2024–2025 will focus on biological staging and include blood tests to improve early, pre-symptomatic identification. IWG pronouncements urge a “clinical-biological” model to maintain clinical context as biomarkers proliferate (Dubois et al. 2024).

### Next, target tau reduction and microglial/inflammatory pathways

Tau remains the main non-amyloid target. The antisense oligonucleotide BIIB080 (IONIS-MAPTRx) decreased cerebrospinal fluid total-tau and phosphorylated tau, showing promising clinical trends in early Alzheimer’s disease. It has progressed to phase 2, suggesting gene-directed tau reduction may be a near-term disease-modifying intervention. The field shifted to epitope re-targeting and earlier intervention after some N-terminal anti-tau antibodies failed (Arastoo et al. 2024).

Innate-immune modulation is progressing, with preliminary studies indicating biomarker-associated signals for soluble TNF (XPro1595) and complement C1q (ANX005). Larger trials will determine efficacy and optimal timing with anti-Aβ or tau therapies (Sakurai et al. 2025).

### Metabolic and vascular techniques may change prevention and early intervention

GLP-1 receptor agonists like semaglutide have strong mechanistic and epidemiological validation and are being evaluated in two large phase-3 trials (evoke/evoke+) with primary-phase completion expected in late 2025. If successful, they would establish a neuro-metabolic class influencing amyloid, tau, inflammation, and neurovascular pathways (Cummings et al. 2025).

SPRINT-MIND 2.0 and related initiatives study long-term dementia outcomes and adverse event trade-offs, while strict blood pressure management reduces mild cognitive impairment and improves cerebrovascular function (Williamson 2025).

### Evidence-based lifestyle, digital, and sensory-stimulation therapies are emerging

The WW-FINGERS global network is increasing multidomain lifestyle interventions and standardized data to assess population-level generalizability and implementation if disease-modifying drugs remain expensive and resource-intensive (Kivipelto et al. 2020).

In phase-2 studies, non-pharmacological neuromodulation, particularly 40-Hz sensory stimulation, showed structural and biomarker signs. Replication with clinical objectives is a priority for 2025–2027 (Sabbagh et al. 2025a, b).

### Additional field requirements

Biomarker-based stopping rules and adaptive designs are expected to favor biologically aligned combination trials (e.g., anti-Aβ induction followed by tau-lowering maintenance, or anti-Aβ with microglial/complement modulators) from 2025 to 2030 (Hodsdon et al. 2025).

Equity-focused diagnostics—validated biomarker-based models across diverse ancestries, comorbidities, and primary care environments—and explicit referral channels are essential to prevent gaps when biologically-defined criteria are integrated into clinical practice (Jack et al. 2024).

Learning health systems that integrate electronics, imaging, biomarker databases, ARIA, and outcome registries (now developing for lecanemab) will translate efficacy into effectiveness, improve risk models (APOE, microbleeds, leukoaraiosis), and enable real-time pharmacovigilance (Amin and Harris 2021).

## Conclusions

Alzheimer’s disease remains one of the most complex and challenging neurodegenerative disorders, with multifactorial causes and limited curative options. Although substantial progress has been made in understanding its molecular basis, most available therapies provide only symptomatic relief. Emerging evidence supports the integration of multi-targeted therapeutic strategies combining pharmacological, lifestyle, and biomarker-based approaches. Future research should focus on early detection, patient-specific precision medicine, and combination regimens addressing amyloid, tau, inflammation, and vascular dysfunction. Advancements in clinical trials, neuroimaging, and genomic profiling are expected to transform AD management, offering renewed hope for disease modification and improved patient outcomes. The current review focuses on bridging the gaps in AD’s current and emerging Therapies.

## Data Availability

All data is available within the manuscript.

## Abbreviations

Aβ: Amyloid-beta
ACh: Acetylcholine
AChEI: Acetylcholinesterase inhibitor
AD: Alzheimer’s disease
ADAM: A disintegrin and metalloprotease
ApoE: Apolipoprotein E
APP: Amyloid precursor protein
ARIA: Amyloid-related imaging abnormalities
BBB: Blood-brain barrier
BACE1: Beta-site amyloid precursor protein cleaving enzyme 1 (Beta-secretase 1)
CAA: Cerebral amyloid angiopathy
cAMP: Cyclic adenosine monophosphate
cGMP: Cyclic guanosine monophosphate
CNS: Central nervous system
CSF: Cerebrospinal fluid
FDA: Food and drug administration
FMT: Fecal microbiota transplantation
FINGER: Finnish geriatric intervention study
GABA: Gamma-aminobutyric acid
GLP-1: Glucagon-like peptide-1
GWAS: Genome-wide association studies
IL-1β: Interleukin-1 beta
mGluR: Metabotropic glutamate receptor
MRI: Magnetic resonance imaging
NFTs: Neurofibrillary tangles
NMDA: N-methyl-D-aspartate
NSAIDs: Nonsteroidal anti-inflammatory drugs
PET: Positron emission tomography
PHFs: Paired helical filaments
PDE: Phosphodiesterase
PDEI: Phosphodiesterase inhibitor
pE-Aβ: Pyroglutamate-modified amyloid-beta
PP2A: Protein phosphatase 2 A
ROS: Reactive oxygen species
SCFA: Short-chain fatty acids
5-TNF-α: Tumor necrosis factor alpha
TREM2: Triggering receptor expressed on myeloid cells 2
